# The Proton-Pump Inhibitor Lansoprazole Enhances Amyloid Beta Production

**DOI:** 10.1371/journal.pone.0058837

**Published:** 2013-03-08

**Authors:** Nahuai Badiola, Victor Alcalde, Albert Pujol, Lisa-Marie Münter, Gerd Multhaup, Alberto Lleó, Mireia Coma, Montserrat Soler-López, Patrick Aloy

**Affiliations:** 1 Institute for Research in Biomedicine. Joint IRB-BSC Program in Computational Biology, Barcelona, Spain; 2 Anaxomics Biotech, Barcelona, Spain; 3 Institut fuer Chemie und Biochemie, Freie Universitaet, Berlin, Germany; 4 Centro de Investigación Biomédica en Red sobre Enfermedades Neurodegenerativas (CIBERNED), Department of Neurology, Hospital de Sant Pau, Barcelona, Spain; 5 Institució Catalana de Recerca i Estudis Avançats (ICREA), Barcelona, Spain; Nathan Kline Institute and New York University School of Medicine, United States of America

## Abstract

A key event in the pathogenesis of Alzheimer’s disease (AD) is the accumulation of amyloid-β (Aβ) species in the brain, derived from the sequential cleavage of the amyloid precursor protein (APP) by β- and γ-secretases. Based on a systems biology study to repurpose drugs for AD, we explore the effect of lansoprazole, and other proton-pump inhibitors (PPIs), on Aβ production in AD cellular and animal models. We found that lansoprazole enhances Aβ37, Aβ40 and Aβ42 production and lowers Aβ38 levels on amyloid cell models. Interestingly, acute lansoprazole treatment in wild type and AD transgenic mice promoted higher Aβ40 levels in brain, indicating that lansoprazole may also exacerbate Aβ production *in vivo*. Overall, our data presents for the first time that PPIs can affect amyloid metabolism, both *in vitro* and *in vivo*.

## Introduction

Alzheimer’s disease (AD) is the most prevalent neurodegenerative disorder worldwide. Many molecular lesions have been detected in AD, but one of the major pathological hallmarks is the extracellular deposition of amyloid-β (Aβ) peptides in the brain [1], that results in oxidative and inflammatory damage, which in turn leads to energy failure and synaptic dysfunction [2]. Formation of Aβ species is caused by the sequential cleavage of amyloid precursor protein (APP) by two proteases, β-secretase, also called β-site APP-cleaving enzyme 1 (BACE1), and γ-secretase. Aβ40 is the major secreted form while Aβ42 has been suggested to be the main pathological species in AD pathogenesis [3], although other truncated Aβ peptides might also contribute substantially to toxicity and amyloidogenesis [4].

Several studies support the hypothesis that classic fibrillar amyloid plaques are deleterious to the brain, showing that the subpopulation of dense-core Aβ plaques in particular, the so-called neuritic plaques, are intimately associated with local dendritic spine loss, changes in neurites, and gliosis in AD and mouse models [5], [6]. However, the total number of amyloid plaques do not correlate well with the severity of illness [7] or with loss of neurons [8], arguing against a direct causal effect of plaques on cognition or neuronal cell death in AD.

More recently, an alternative hypothesis has been growing and gaining support, based on the idea that the toxic component is within the soluble fraction. There is data showing that soluble forms of Aβ correlate more closely with dementia severity than fibrillar Aβ [9], [10], [11], [12] and that Aβ oligomers alter dendritic spine density and affect hippocampal synaptic plasticity *in vivo* [13], [14], [15], [16], [17]. Furthermore, it has been demonstrated that brain oligomeric Aβ, but not total amyloid plaque burden, correlates with neuronal loss and astrocyte inflammatory response in APP/Tau transgenic mouse model [18]. In this context, several studies show the presence of Aβ oligomers in CSF of AD patients, while not in healthy individuals, and also the direct correlation of oligomers with cognitive impairment [19], [20], [21].

Despite all the efforts put on AD research over the past years, there are no effective treatments to prevent, halt or cure the disease. Indeed, there are only four FDA-approved drugs for AD treatment, although they mainly provide a symptomatic improvement and are unable to stop the disease progression [22]. This prompted us to use the therapeutic performance mapping system (TPMS) [23] to explore potential novel indications of marketed drugs to modify the biology of AD. In brief, the TPMS is a top-down systems biology approach with potential applications in drug repositioning [24]. Starting from the clinical effects produced by different therapeutic compounds (both, positive and negative), we first split them into causative physiological motifs and identified the responsible molecular effectors, which were then mapped onto the disease-related cell network. We afterwards established different relationships between drug targets and effector proteins in the network that are used for training a classifier, with capacity for predicting and scoring novel potential indications on AD of totally unrelated drugs. The complete results of this study will be published elsewhere [25]. Interestingly, the TPMS analysis suggested that lansoprazole could act as a strong potential modulator of the processes involved in amyloid-β pathology.

Lansoprazole is a proton pump inhibitor (PPI) drug widely used in the treatment of peptic ulcer disease and other conditions where inhibition of gastric secretion may be beneficial [26], [27]. PPIs are generally well tolerated, and adverse effects are relatively infrequent [28], [29]. Yet, chronic administration of PPIs is becoming increasingly common, and there is a growing concern about potential unexplored adverse effects from such long-term therapy [30].

In this study, we explore the effects of lansoprazole, and other PPIs, on β-amyloid production in a well-established cellular model of amyloid pathology, with special attention to the effect over the different Aβ species. We assess the *in vivo* relevance of our findings in wild-type (wt) and AD triple transgenic (3xTg-AD) mice and we ultimately speculate about the potential mechanisms underlying the observed alterations.

## Materials and Methods

### Cell Culture

We maintained Chinese hamster ovary (CHO) cells, stably transfected with both wild-type (wt) human APP and PS1 (PS70 cells; [31]), in Dulbecco's modified Eagle's medium (DMEM) (GIBCO® Life Technologies) supplemented with 10% fetal bovine serum (GIBCO®), 25 µg/mL of puromycin and 200 µg/mL of G418 antibiotics (Sigma-Aldrich).

### Drug Treatments

For Aβ production analysis, we seeded PS70 cells in 12-well plates at a density of 150,000 cells per well and we subsequently treated for 24 h with lansoprazole, omeprazole, pantoprazole or esomeprazole (Sigma-Aldrich) at different concentrations. We used the γ–secretase inhibitor DAPT (N-[N-(3,5-Difluorophenacetyl)-L-alanyl]-S-phenylglycine t-butyl ester, Sigma-Aldrich) at 2 µM as a positive control. Lastly, we collected and stored cell culture supernatants at −80°C until further use.

### Analysis of Aβ Peptides by ELISA

We quantified human Aβ42 and Aβ40 levels by human β-amyloid (1–42) and (1–40) ELISA kits (Wako Pure Chemical Industries), respectively. We loaded 100 µL of conditioned media on every assay. For 3xTg-AD mouse brain extracts we previously diluted them 1/40 in standard buffer and then we loaded following manufacturer’s protocol. We measured rodent Aβ40 and Aβ42 levels with ELISA kits (IBL International) according to manufacturer’s protocol.

### Mass Spectrometry of Aβ Species

We used W0-2antibody and Protein-G Sepharose beads to immunoprecipitate human Aβ from conditioned media. We washed Sepharose beads twice in PBS and twice in 100 mM ammonium acetate. We then eluted Aβ twice with 300 µl of 50% acetic acid and vacuum-dried. Finally, we resuspended the samples in 10 µl of 33% acetonitrile containing 0.1% tri-fluoric acetic acid and ultrasonicated. Afterwards we analyzed Aβ species by MALDI-MS on sinapinic acid matrix with an UltraflexII TOF/TOF (BrukerDaltonics).

### Animals and Treatments

All animals were housed in an animal facility that is fully compliant with the European policy on the use of Laboratory Animals. Experimental protocols were approved by the Parc Científic of Barcelona Committee and meet the European and Spanish guidelines of animal experimentation.

We treated both female 3xTg-AD mice (Charles River) and non-transgenic mice (B6129SF1/J) at 7 month of age for 5 consecutive days with an intraperitoneal injection of lansoprazole. We diluted and administered lansoprazole in 10% DMSO and 18% of encapsin (2-hydroxipropil beta-cyclodextrine), at 20 mg/kg or 100 mg/kg, respectively. At the end point, we sacrificed mice 5 h after the last treatment and we froze each hemisphere in liquid nitrogen and stored them at −80°C.

### Brain Soluble Aβ Extraction

We thawed non-transgenic mouse brains on ice in 3× (w/v) 0.2% of diethylamine (DEA) and 50 mM of NaCl buffer with a protease inhibitor cocktail (Complete® EDTA-free, Roche), and then we homogenized them. We subsequently centrifuged homogenates at 100.000 g for 1 h at 4°C. Finally, we collected the supernatants, and neutralized them by addition of 1∶10 volume of 0.5 M Tris-HCl pH 6.8. We stored samples at −80°C as DEA-soluble Aβ fractions.

We thawed 3xTg-AD mouse brains on ice in 3× (w/v) 2% SDS with a protease inhibitor cocktail (Complete® EDTA-free, Roche) and then we homogenized them. Next, we centrifuged homogenates at 100.000 g for 1 h at 4°C. Finally, we collected the supernatants and we stored samples at −80°C as SDS-soluble Aβ fractions.

### Western Blotting

We determined lysate protein concentration using the Bio-Rad D_C_ protein assay (Bio-Rad Laboratories). We loaded 20–40 µg of each cell lysate and electrophoresed in 10% Tris-glycine gels for Western blot analysis. For Aβ species detection, supernatants we run in 11% urea gels. For the immunoblotting we incubated overnight at 4°C with the following primary antibodies: rabbit polyclonal anti-C-terminal APP (Sigma-Aldrich), rabbit polyclonal anti-BACE (catalytic domain, Abcam), rabbit polyclonal anti-sAPPβ (IBL), 6E10 (against Aβ 1–16, Covance) or mouse monoclonal anti-actin (Sigma-Aldrich) antibodies. Afterwards, we incubated with either an HRP-conjugated secondary antibody plus enhanced chemiluminescence substrate (Millipore), or with an infrared fluorescent-labelled secondary antibody (IRDye, Rockland Immunochemicals, Gilbertsville, PA) for 1 h at room temperature.

### Statistical Analysis

All data are shown as mean ± SD. Statistical tests included one-way ANOVA for repeated measures and t-test when appropriate.

## Results and Discussion

### Lansoprazole and Other PPIs Increase Aβ Levels in AD-like Cellular Models

**Figure 1:**
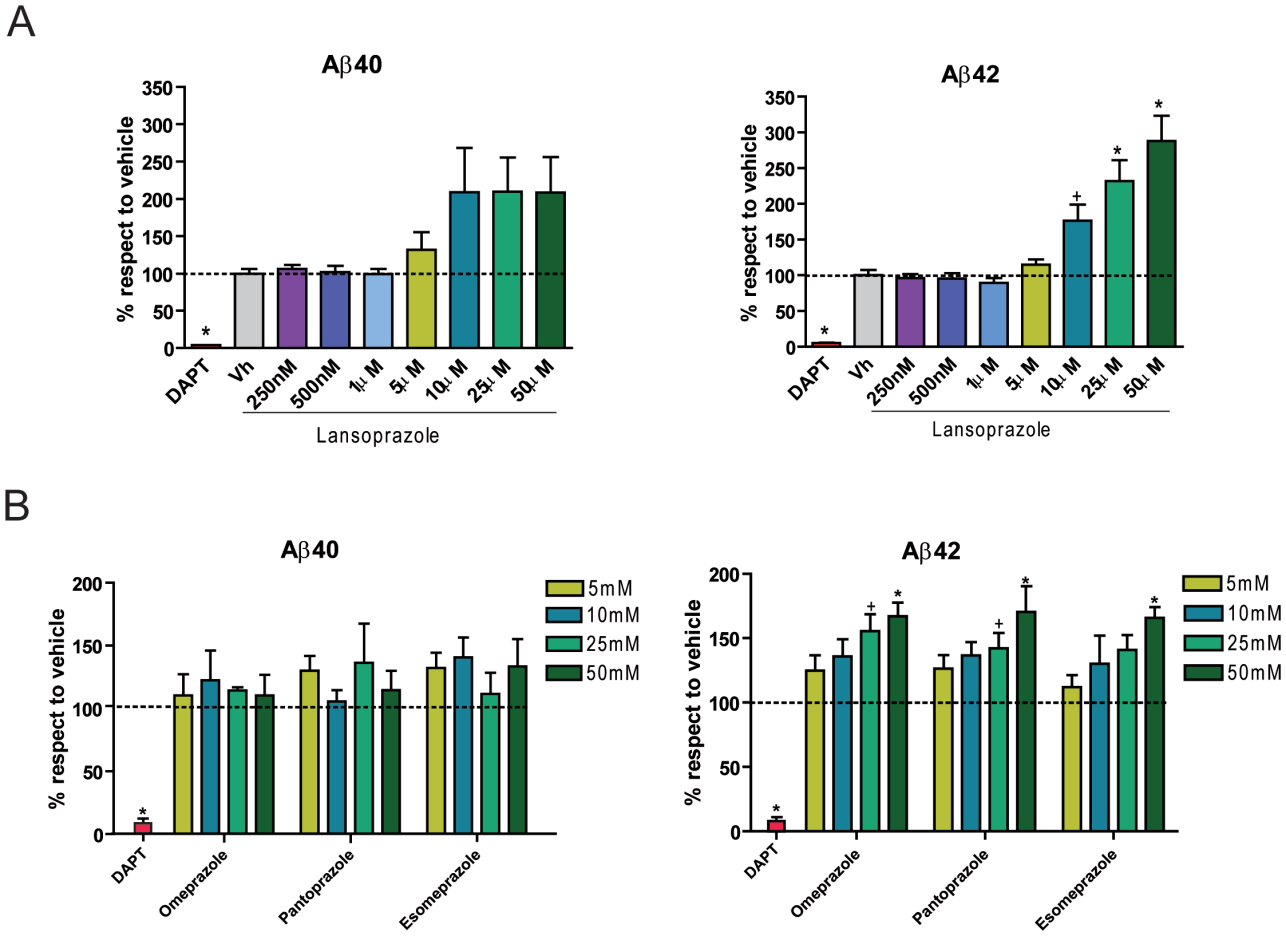
Lansoprazole and similar PPIs increase Aβ levels at 5 µM–50 µM range in AD-like cells. **A**, Treatment of PS70 cells with lansoprazole at different concentrations (250 nM–50 µM) for 24 h increased Aβ40 and Aβ42 levels, as measured by ELISA immunoassays (n = 6± SD) p<0.05 (+), p<0.01 (*). **B**, Similar treatment with omeprazole, pantoprazole and esomeprazole at different concentrations (5 µM–50 µM) also increased Aβ40 and Aβ42 levels (n = 4± SD) p<0.05 (+), p<0.01 (*).

To investigate the potential effect of lansoprazole on Aβ production, we treated PS70 Chinese hamster ovary (CHO) cells stably expressing both wild-type human APP and presenilin 1 (PS1) with increasing concentrations of lansoprazole for 24 h. We first used the MTT reduction assay as a proxy to determine whether the tested concentrations caused cell toxicity, which was not the case below 50 µM (data not shown), in agreement with previous studies [32]. We subsequently measured the Aβ40 and Aβ42 levels in conditioned media of cultures by specific ELISA immunoassays. We observed that Aβ40 levels in the conditioned media increased after treatments above 10 µM (Figure 1A), up to 2-fold increase in the amount of Aβ40 with respect to the vehicle-treated cultured cells. Furthermore, we observed a dose-dependent increase in Aβ42 between 5 µM and 50 µM (Figure 1A), with more than 200% Aβ42 increase at 25 µM and over 300% when treated with 50 µM. As expected, cells treated with the γ–secretase inhibitor N-[N-(3,5-Difluorophenacetyl)-L-alanyl]-S-phenylglycine t-butyl ester (DAPT) showed negligible levels of Aβ. These results indicate that lansoprazole treatment increase Aβ production at commonly used concentrations [32], [33], [34], [35], [36], which in turn are comparable to other Aβ inducers such as the calcium ionophore A23187, which increases the production of Aβ approximately 3-fold [37], or caffeine (at millimolar concentrations), that increases Aβ levels until 4-fold [38]. In addition, we have observed an Aβ42 increase of over 250% compared to vehicle in cell cultures treated at 50 µM lansoprazole, while fenofibrate, a potent Aβ42 raising Aβ38 lowering compound, generates an increase of 125% at the same concentration [39]. Overall, these findings provide further evidence about the accuracy of the TPMS to predict pharmacological alterations in Aβ metabolism.

To check whether these results could also be extrapolated to other PPIs, we examined the effect of compounds of the same drug class on Aβ production. Omeprazole is one of the most widely prescribed drugs worldwide, which is administered as a racemic mixture of its two enantiomers, S-omeprazole (esomeprazole) and R-omeprazole. Similarly, pantoprazole is also broadly used because of its relatively long duration of action compared with other PPIs, and its lower propensity to become activated in slightly acidic body compartments [40]. Since the chemical structures and pharmacological targets of the four PPIs are very similar, our TPMS computational analyses also predicted an effect of these compounds on the Aβ metabolism. Thus, we decided to investigate the effect of omeprazole, pantoprazole and esomeprazole within 5 µM and 50 µM range, based on our previous results. Although the increase in Aβ40 was non-homogeneous for the 3 drugs, they did show a dose-dependent increase in Aβ42 levels (Fig. 1B), achieving up to 175% increase. Hence, these results suggest that the modulation of Aβ production is a shared feature among the PPI drug class.

### Lansoprazole Might Modulate the γ-secretase Complex

**Figure 2:**
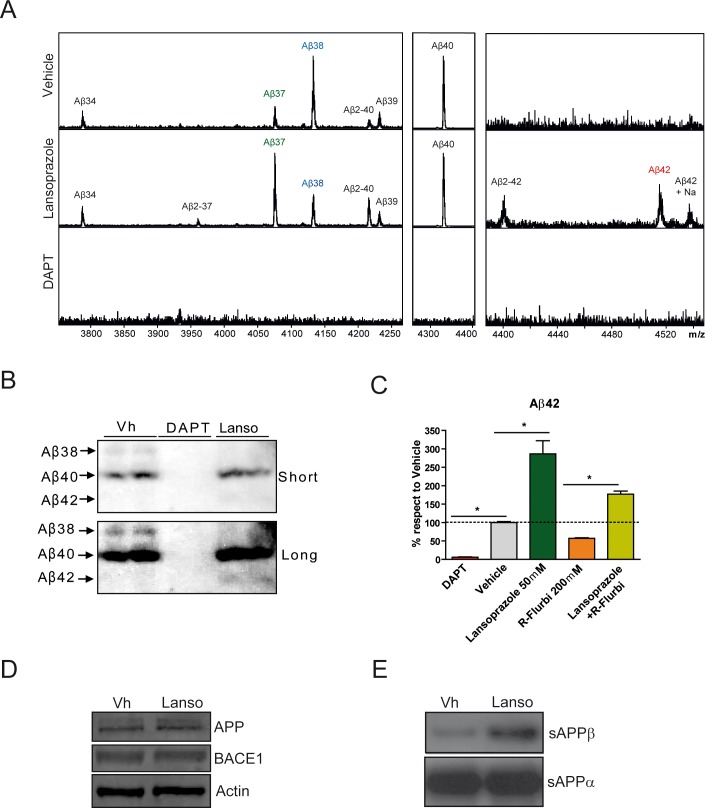
Lansoprazole changes the production levels of several Aβ species. **A**, MALDI-MS analysis of Aβ-immunoprecipitated species from conditioned PS70 supernatants. Cells treated with lansoprazole at 50 µM for 24 h showed a significant increase on Aβ42 compared to vehicle spectrum. DAPT treated cells were used as a negative control, showing none Aβ species as expected. **B**, Western blot analysis of the different Aβ species present in treated PS70 supernatants. A decrease on Aβ38 and increase on Aβ42 was detected in conditioned media of treated cells with lansoprazole at 50 µM for 24 h. Short and long exposures are shown for better visualization. **C**, Aβ40/42 levels in conditioned media from treated PS70 cells were measured by ELISA immunoassays (n = 3± SD) p<0.01 (*). Cells treated with both lansoprazole at 50 µM and R-flurbiprofen at 200 µM for 24 h displayed reduced Aβ42 levels when compared to lansoprazole-only treated cells, indicating the lansoprazole is capable to counteract the R-flurbiprofen Aβ42 lowering effect. **D**, Western blot analysis of APP and BACE1 protein levels in total lysates, showing no differences between treated and non-treated cells. A representative experiment is shown (n = 3 independent experiments). **E**, Western blot analysis of sAPPβ and sAPPα protein levels in conditioned media. The immunoblot shows an increase in sAPPβ in conditioned media from cells treated with lansoprazole. A representative experiment is shown (n = 3 independent experiments).

To gain a deeper insight in the relative formation of Aβ species induced by lansoprazole, we conducted an Aβ-immunoprecipitation coupled with mass spectrometry analysis of high dose lansoprazole-conditioned media. Different Aβ species are generated because γ–secretase has multiple APP cleavage sites. The main produced species is Aβ40 and, to a lower extent, Aβ38 and Aβ42. Intriguingly, small molecule drugs called γ–secretase modulators (GSM) are able to shift the γ–secretase cleavage site, being classified as straight GSMs when they lower Aβ42 and rise Aβ38 [41], or as inverse GSMs (iGSM) when they do the opposite [39], [42], [43]. Interestingly, our MALDI-MS analysis revealed a considerably altered Aβ peptide pattern in cells treated with lansoprazole at 50 µM. The relative levels of Aβ42 increased whereas the relative levels of Aβ38 decreased (Figure 2A). Intriguingly, there was also an increase in Aβ37. As expected, the γ-secretase inhibitor DAPT completely abrogated Aβ production, showing no Aβ peaks. We further confirmed the Aβ42 increase and Aβ38 decrease by Western blot (Figure 2B). Therefore, attending to the results obtained with lansoprazole in the Aβ species production shift, one possible explanation would be that lansoprazole might act as an inverse GSM.

To further investigate this hypothesis, we wanted to test if lansoprazole was able to counterbalancing the Aβ42-lowering capability of R-flurbiprofen, a well characterized non-steroidal anti-inflammatory drug (NSAID) that acts as straight GSM [44], [45]. NSAIDs are widespread used due to the prevalence of diseases in the aging population and to their crucial role as effective antipyretic analgesics in a wide spectrum of conditions and diseases ranging from a common cold to rheumatoid arthritis [46]. However, they are known to disrupt the mucosal resistance to gastric acid through several mechanisms including suppression of prostaglandin production and are thus associated with adverse events such as gastric or duodenal ulcers. For that reason, the co-administration with PPIs is strongly recommended in certain circumstances [47], [48]. Notably, albeit we can find straight and inverse GSM modulators within NSAIDs, the former have been considered as very interesting therapeutic agents in AD, since they can lower Aβ42 without perturbing the cyclooxygenase (COX) activity, the principal pharmacological target of NSAIDs [41], [49].

As expected, cells treated with R-flurbiprofen showed decreased Aβ42 levels while cells treated with lansoprazole increased Aβ42 levels (Figure 2C). Interestingly, when the two drugs were combined, lansoprazole blocked the Aβ42 decrease induced by R-flurbiprofen. Therefore, these results suggest that concomitant administration of PPIs with NSAIDs may neutralize the Aβ42 lowering effect provided by NSAIDs, at least in cell culture.

Taken together, these findings suggest that lansoprazole increase Aβ42 production similarly to other described iGSMs. Nevertheless, alternative mechanisms related to APP dimerization processes could also play a role in the observed changes of Aβ42 levels [50].

### Lansoprazole Might Increase BACE1 and Meprin β Protease Activities

The putative iGSM activity of lansoprazole could explain the increase of Aβ42 coupled to a decrease of Aβ38, but it cannot account for the rise of Aβ40. Hence, we wanted to investigate other possible effects of lansoprazole on Aβ production. The APP can be processed by two different pathways: the non-amyloidogenic pathway, involving α-secretase and γ-secretase activities, and the amyloidogenic pathway, requiring BACE1 and γ-secretase [2]. If the first cleavage is performed by α-secretase, soluble APPα (sAPPα) and APP C83 C-terminal fragments are generated, and the consecutive cleavage by γ-secretase produces the p3 peptides, which are non-amylodogenic. On other hand, when APP is first cleaved by BACE1, soluble APPβ (sAPPβ) and APP C99 fragments are otherwise generated, and γ-secretase cleavage ultimately generates Aβ peptides and the amyloid precursor protein intracellular domain (AICD) fragments.

To explore how lansoprazole was able to increase Aβ37 and Aβ40 levels, we first tested whether it increased APP or BACE1 protein levels, since higher amounts of Aβ substrate or processing enzyme would certainly explain the increase in Aβ production [1], [51]. However, we did not observe any significant change in the protein expression levels of either protein (Figure 2D). We then interrogated if lansoprazole could enhance BACE1 activity instead, and measured the generation of sAPPβ, a BACE1 cleavage product. Interestingly, we observed that lansoprazole promoted sAPPβ production, suggesting an increase of BACE1 activity. In contrast, sAPPα remained unaffected by lansoprazole treatment (Figure 2E).

In addition, the mass spectra also revealed a slight increase of the Aβ2-37, Aβ2-40 and Aβ2-42 species (Fig. 2A), which could be accounted for the meprin β metalloprotease, recently identified as an APP cleaving enzyme at the p2 position [52]. Variations in the media pH induced by lansoprazole could thus boost this protease activity, generating Aβ2-x peptides.

Overall, these findings suggest that lansoprazole may not only modulate γ-secretase, but also seems to increase BACE1 activity, boosting the Aβ species production that would be eventually reflected in Aβ40 and Aβ37 increased levels. Nevertheless, since the PPIs are known to be irreversible inhibitors of H^+^/K^+^ ATPase, lansoprazole-induced variations in the media pH may also affect the activity of other proteases, such as meprin β, and ultimately affecting Aβ production, particularly Aβ2-x species.

### Acute Treatment with Lansoprazole Increases Aβ Levels in Both wt and AD Mouse Models

**Figure 3:**
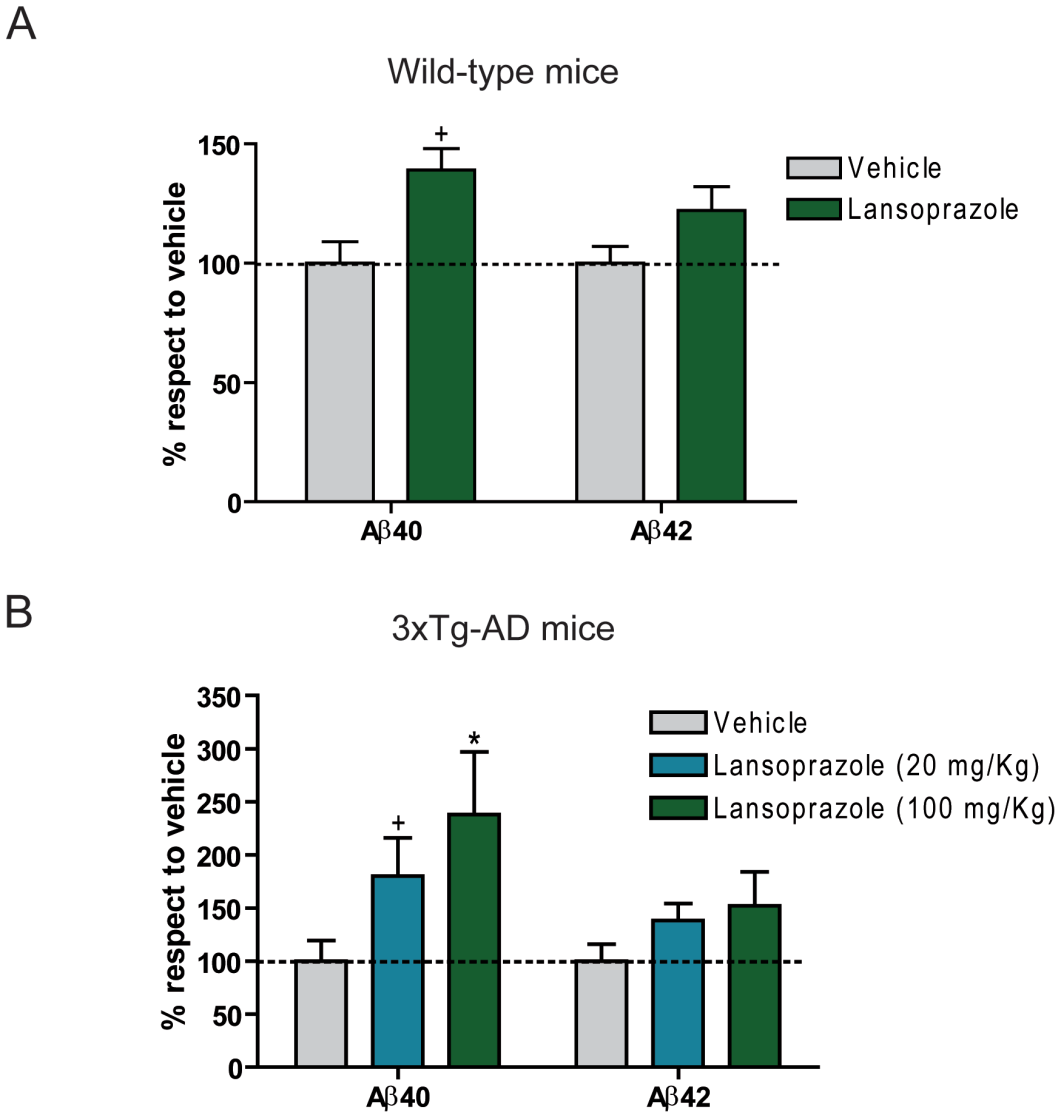
Lansoprazole raises Aβ40 production in mice. **A**, Non-transgenic mice were treated 5 consecutive days with 100 kg/mg of lansoprazole (n = 10). Soluble Aβ40 and Aβ42 from brain extracts were measured by ELISA (n = 10± SD) p<0.05 (+). Lansoprazole increased Aβ40 levels in non-transgenic mice. B, 3xTg-ADwere treated 5 consecutive days with 100 kg/mg of lansoprazole (n = 6). Soluble Aβ40 and Aβ42 from brain extracts were measured by ELISA (n = 6± SD) p<0.05 (+), p<0.01 (*). Lansoprazole increased soluble Aβ40 levels in 3xTg-AD mice in a dose-dependent manner.

Our results would not go beyond a cellular curiosity if lansoprazole could not cross the blood brain barrier. However, it has been reported that it can indeed cross it and exert its effects in brain tissue [53]. Thus, to determine whether lansoprazole is capable of altering Aβ production in the brain, we conducted short-term intraperitoneal administration in wt and AD triple-transgenic (3xTg-AD) mouse models, like in previous studies [39] (Figure 3). 3xTg-AD mice overexpress human tau and APP in a mutant PS1 knock-in background, and present both plaque and tangle pathologies in an age-related manner [54]. Even though, acute treatments do not enable an evaluation of histopathological or cognitive alteration, since changes in Aβ plaque burden or cognitive impairment usually occur after a long and sustained treatment of at least 2–3 months. Yet, 8-month age transgenic mice typically contain few Aβ plaques but they have significant amounts of intracellular and soluble Aβ [54], being a suitable age to test changes in soluble Aβ.

We used administration doses of 20 mg/kg/day and 100 mg/kg/day. When compared to the equivalent doses in human [55], the administered concentrations are far below the LC50 (5000 mg/Kg) and comparable to the doses prescribed to treat certain pathologies, such as the Zollinger-Ellison syndrome, 180 mg/day [56]. We found that, indeed, acute treatment with 100 mg/kg/day intraperitoneally administered lansoprazole increased soluble Aβ40 levels in healthy, non-transgenic mice (Figure 3A). Levels of soluble Aβ42 were also slightly increased, although they did not reach statistical significance. In the case of 3xTg-AD mice, short-term treatment with 20 mg/kg/day or 100 mg/kg/day of lansoprazole dramatically raised soluble Aβ40 levels in a dose-dependent manner (Figure 3B). Interestingly, Aβ40 production was higher than in non-transgenic mice, attaining almost to the 250% rise at 100 mg/kg/day. Similarly to the non-transgenic mice, we also observed a moderate increase in soluble Aβ42 levels, although they were not statistically significant either. In order to contextualize the relevance of the effects shown in mice at doses used in this study, it is worth to mention that a recent patent proposes a daily dose up to 400 mg of lansoprazole to inhibit tumor growth in humans [33]. Using the body surface area (BSA) normalization method [55], the conversion of the 20 mg/Kg and 100 mg/Kg mouse doses into equivalent human doses results in around 100 mg/day and 486 mg/day, respectively. Interestingly, we found an increase of Aβ levels in 3xTg-AD mice treated with 20 mg/Kg/day of lansoprazole, providing evidence of a clear effect at human prescribed equivalent doses.

The differences observed in the Aβ40/Aβ42 levels between cellular and mouse models could be partially explained by the Aβ quantization, since we only measured extracellular Aβ in cells, while we measured both extracellular and intracellular Aβ species in brain homogenates. Despite these differences, our findings demonstrate that lansoprazole is able to augment Aβ production both *in vitro* and *in vivo* models, with an exacerbated effect in AD models.

### Conclusions

Our results reveal that lansoprazole, in addition to its known inhibitory effect on gastric acid production, has an effect on Aβ generation. Although the underlying mechanisms remain elusive, our observations show that lansoprazole increases Aβ37, Aβ40 and Aβ42 and lowers Aβ38 levels in an AD-like cell model. In addition, the increase of sAPPβ and the lack of changes in APP and BACE1 protein levels seem to indicate that lansoprazole would not only modulate the γ-secretase complex, but also increase BACE1 activity.

**Figure 4:**
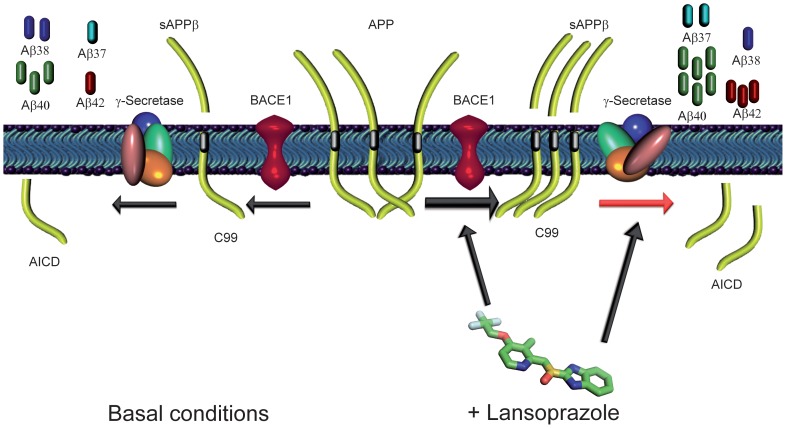
Hypothetical mechanisms of lansoprazole on Aβ production. Aβ peptides are produced from the consecutive cleavage of APP by BACE1 (β-secretase) and γ-secretase. The first cleavage generates soluble APPβ (sAPPβ) and the C99 C-terminal fragment, while the subsequent one releases Aβ peptides and the amyloid precursor protein intracellular domain (AICD). In basal conditions (left), a variety of Aβ species are formed. Conversely, when cells are treated with lansoprazole (right), BACE1 activity could be increased, generating more sAPPβ and C99 fragments and subsequently increasing the overall Aβ production. Lansoprazole also could act as an inverse GSM, shifting the γ–secretase cleavage, augmenting Aβ42 and reducing Aβ38. Together, lansoprazole is able to increase Aβ37, Aβ40 and Aβ42 species and decrease Aβ38.

Taken together, we hypothesize that lansoprazole could inversely modulate the γ-secretase activity by shifting the APP cleavage site, resulting in higher Aβ42 and lower Aβ38 levels (Figure 4). Moreover, it might also increase the activity of other pH-dependent proteases, such as BACE1, raising total Aβ production and particularly reflected in the raise of Aβ37 and Aβ40 levels, or meprin β, boosting Aβ2-x species. Nevertheless, further experiments are needed to better understand the role of lansoprazole in Aβ production and specifically to unveil its underlying mechanisms.

Notwithstanding, from a more clinical perspective, since PPIs are commonly used drugs, it would be interesting to perform epidemiologic studies to investigate whether the long-term use of PPIs could have any detrimental impact on AD, particularly in aged chronic recipients. Recent studies have actually reported potential inappropriate prescriptions (PIM) in aged people with dementia [57], where PPIs appeared among the most prevalent PIMs when used at maximum therapeutic dosage for more than 8 weeks [57].

We believe that our data demonstrate for the first time that lansoprazole and other PPIs can increase Aβ not only in cell cultures but also in mice. These results can serve as a catalyst for further studies in order to evaluate whether the treatment with PPIs may have an impact on AD pathology.
